# Bisphenol A exposure and type 2 diabetes mellitus risk: a meta-analysis

**DOI:** 10.1186/s12902-018-0310-y

**Published:** 2018-11-06

**Authors:** Semi Hwang, Jung-eun Lim, Yoonjeong Choi, Sun Ha Jee

**Affiliations:** 1 Department of Epidemiology and Health Promotion, Institute for Health Promotion, Graduate School of Public Health, Yonsei University, 50-1 Yonsei-ro, Seodaemun-gu, Seoul, 03722 Republic of Korea; 2 Department of Public Health, Graduate School, Yonsei University, Seoul, Republic of Korea

**Keywords:** Bisphenol a (BPA), Endocrine disrupting chemicals (EDCs), Diabetes mellitus (DM), Type 2 diabetes mellitus (T2DM), Hemoglobin A1c (HbA1c), Fasting plasma glucose, Obesity, Meta-analysis

## Abstract

**Background:**

This meta-analytic study explored the relationship between the risk of type 2 diabetes mellitus (T2DM) and bisphenol A concentrations.

**Methods:**

The Embase and Medline (PubMed) databases were searched, using relevant keywords, for studies published between 1980 and 2018. A total of 16 studies, twelve cross-sectional, two case-control and one prospective, were included in the meta-analysis. The odds ratio (OR) and its 95% confidence interval (CI) were determined across the sixteen studies. The OR and its 95% CI of diabetes associated with bisphenol A were estimated using both fixed-effects and random-effects models.

**Results:**

A total of 41,320 subjects were included. Fourteen of the sixteen studies included in the analysis provided measurements of urine bisphenol A levels and two study provided serum bisphenol A levels. Bisphenol A concentrations in human bio-specimens showed positive associations with T2DM risk (OR 1.28, 95% CI 1.14, 1.44). A sensitivity analysis indicated that urine bisphenol A concentrations were positively associated with T2DM risk (OR 1.20, 95% CI 1.09, 1.31).

**Conclusions:**

This meta-analysis indicated that Bisphenol A exposure is positively associated with T2DM risk in humans.

**Electronic supplementary material:**

The online version of this article (10.1186/s12902-018-0310-y) contains supplementary material, which is available to authorized users.

## Background

Type 2 diabetes mellitus (T2DM) is a metabolic disease that presents with symptoms of insulin resistance and lack of insulin [1]. The global prevalence of T2DM among adults is about 415million, but based on projections by the International Federation of Diabetes is expected to reach 642 million in 2040 [2, 3].

Bisphenol A (BPA) is a role for endocrine disrupting chemicals (EDCs) and especially used in epoxy resin and polycarbonate plastic products such as food packaging, drink containers, and dental sealants [4–7]. Once the EDCs is deposited in the body, they can interfere with the physiological effects of estrogen, androgen and thyroid hormones by functioning as a hormone agonists and antagonists. Especially, BPA or EDCs interfere with cell signal pathways related to weight and glucose homeostasis. A number of previous experimental and epidemiological studies have found that EDCs can penetrate the body in several ways, including dietary intake, inhalation, skin contact, and other pathways. Thus, EDCs may have been associated with mainly the occurrence of hormone-like effects disorders and even cancers [7].

Competitive with 17- beta estradiol (E2), BPA is a type of endocrine disrupting chemical (EDCs) that disrupts estrogenic response by binding to estrogen receptors. BPA binds to androgen receptors and thyroid receptors. Unfortunately, humans are exposed to BPA through the daily exposure to BPA containing products such as canned food, plastic products, dental sealants, and household dust [7, 8].

In recent studies, research findings suggest that low levels of BPA can cause significant health problems. A number of scientists hypothesized that adverse health effects might be associated with high urinary BPA concentrations. Epidemiological studies have been carried out to evaluate the possible association between BPA exposure and the risk of T2DM, but the results were not consistent [9–24].

In this study, a meta-analysis focusing on the association between BPA concentrations (measured in urine or serum) and the risk of T2DM was performed. In addition, subgroup analyses were performed according to the sample type (urine or serum) and the study design.

## Methods

### Study selection

**Fig. 1:**
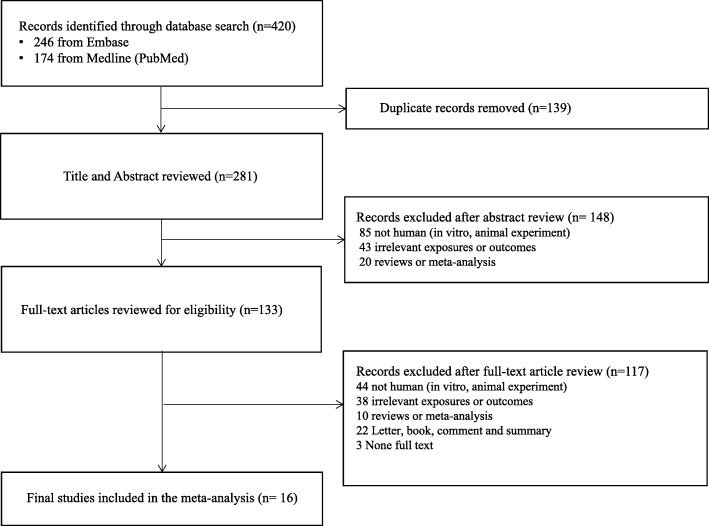
A PRISMA flow diagram

Figure 1 shows a PRISMA flow diagram that describes the selection process of this meta-analysis (Additional file 1: Table S1). As shown in the figure, the Embase and Medline (PubMed) databases were searched between 1980 and 2018 using Medical Subject Headings (MeSH) terms related to BPA and diabetes.

The keywords used in the Embase and Medline (PubMed) database searches were: Bisphenol A, BPA, 4, 4 isopropylidenediphenol or Bisphenol A bis (2 hydroxypropyl) ether dimethacrylate and Noninsulin dependent diabetes mellitus or Type 2 diabetes or Diabetes Mellitus, Type 2 or Diabetes Mellitus, Noninsulin-Dependent or Diabetes Mellitus, Ketosis-Resistant or Diabetes Mellitus, Fasting blood sugar or Fasting plasma glucose or Blood glucose, HbA1c or Glycosylated hemoglobin or Hemoglobin A1cor Glycated Hemoglobin A or Hemoglobin A, Glycated. A total of 420 articles were found: 246 were from Embase and 174 were from Medline (PubMed). First, 139 duplicated articles were removed., After, an initial review, 148 studies were excluded; 85 studies were not human research such as animal and invitro experiment, 43 studies had irrelevant exposures or outcomes and 20 studies were reviews or meta-analyses papers. Next, 133 studies were selected for full-text article review. From these studies, 117 studies, including 44 studies were not human research, 38 with irrelevant exposures or outcomes, 10 studies were reviews or meta-analyses, 22 were letter or book or comment papers, and 3 had not find full text from the same database were excluded. Finally, a total of 16 articles were included in this meta-analysis (Fig. 1).

### Data extraction

Data extraction was completed twice by two reviewers, Hwang, S. and Lim, J.E. independently, with no disagreement in the selection of the final sixteen articles [9–24]. The reviewers selected the variables while considering authors, year of publication, country, type of study, type of sample, unit of measurement, population, comparison categories, and adjusted odds ratios (OR) with corresponding confidence intervals, and model adjustments. To be included in the meta-analysis, a published study had to be the original article published between 1980 and 2018. A total of 16 studies published between September, 2008 and January, 2018 were selected for final inclusion. We conducted quality assessment using the Downs and Black score [25]. The average quality score was 16 with scores ranging from 13 to 18.

### Statistical analyses

Odds ratios (OR) and 95% confidence intervals (95% CI) were obtained from the selected articles using the standard guidelines for meta-analysis [26]. Fixed-effects model and random-effects model were implemented. Heterogeneity was tested using the Cochrane Q-test and I^2^ statistic, considering an I^2^ value > 50% as indicative of substantial heterogeneity. A study with a significantly high OR was omitted from the meta-analysis to avoid overrepresentation. Analyses were performed by sub-groups: type of sample (serum or urine), and type of study (cross-sectional, case-control and prospective) as possible sources of heterogeneity. A Begg’s Funnel Plot and an Egger’s Regression Test were conducted to minimize publication bias and asymmetry of the studies. When publication bias exists, the Begg’s Funnel Plot is asymmetric, or the Egger’s Test *P*-value < 0.05 [27].

To adjust for the cross-study differences between the BPA concentration units and the range of measured values, a dose-response meta-analysis (DRMA) was implemented. The dose-response meta-analyses (DRMA) was implemented by using the STATA GLST command [28] on a sample off our studies (Additional file 2: Figure S2).

Statistical analyses were performed using STATA version 13.0 software (Stata Corp, College Station, Texas).

## Results

The 420 studies were searched using a systematic search strategy, referring to the PRISMA flow chart that describes the selection process of the meta-analysis [29]. After the duplicate records were removed, each article was reviewed by title, abstract, and full-text. Sixteen studies, 12 cross-sectional, 3 case-control and 1 prospective studies remained. A total of 6855 diabetic patients from among 141,320 subjects were included in the study.

Table 1 represents the characteristics of the studies included in the meta-analysis. The selected studies were performed in the USA, Korea, Iran, China and Thailand. While using funnel plot asymmetry to detect publication bias and applying Egger’s regression test to measure for asymmetry, a very low publication bias was confirmed .

**Table 1:** Risk estimates and study information from abstracts of original studies on BPA concentration and type 2 diabetes mellitus

Reference	Country	Type of study	Used sample	Unit	Population (Case / Total)	Comparison categories	Adjusted OR	95% CI	Adjustment in model	Quality score
Lang et al. (2008) [9]	The United States	Cross-sectional	Urine	ng/mL	136 / 1455	BPA continuous	1.39	1.21–1.60	Age, sex, race/ethnicity, education, income, smoking, BMI, waist circumference and urinary creatinine concentrations,	17
Melzer et al. (2010) [10]	The United States	Cross-sectional	Urine	ng/mL	277 / 2947	BPA continuous	1.24	1.10–1.40	Age, sex, race/ethnicity, education, income, smoking, BMI, waist circumference, and urinary creatinine concentration.	18
Silver et al. (2011) [11]	The United States	Cross-sectional	Urine	ng/mL	540 / 4389	BPA continuous	1.08	1.02–1.16	Age, age^2^, urinary creatinine as natural splines (restricted cubic splines) with 4 degrees of freedom (knots at 25th, 50th, and 75th percentiles), BMI, waist circumference, and smoking status.	17
Ning et al. (2011) [12]	The United States	Cross-sectional	Urine	ng/mL	1087 / 3423	BPA in quartiles Q1: ≤0.47, Q2: 0.48–0.81, Q3: 0.82–1.43, Q4: > 1.43	1.37	1.08−1.74	Age, sex, educational level, family history of diabetes, WC, systolic blood pressure, ln(TG level), ln(hsCRP level), ln(ALT level), estimated glomerular filtration rate, albumin level and total bilirubin level.	15
Shanker & Teppala (2011) [13]	The United States	Cross-sectional	Urine	ng/mL	467 / 3967	BPA in quartiles Q1: < 1.10, Q2: 1.10–2.10, Q3: 2.11–4.20, Q4: > 4.20	1.68	1.22–2.30	Age (years), gender, race-ethnicity (non-Hispanic whites, non-Hispanic blacks, Mexican-Americans, others), education categories (below high school, high school, above high school), smoking (never, former, current), alcohol intake (never, former, current), BMI (normal, overweight, obese), systolic and diastolic blood pressure (mm Hg), urinary creatinine (mg/dl), and total cholesterol (mg/dl).	16
Wang et al. (2011) [14]	China	Cross-sectional	Urine	ng/mL	1048 / 3390	BPA in quartiles Q1: ≤0.47, Q2: 0.48–0.81, Q3: 0.82–1.43, Q4: > 1.43	1.37	1.06–1.77	Age, sex, BMI, urinary creatinine concentration, smoking, alcohol drinking, education levels, systolic blood pressure, HDL-C, LDL-C, TC, TG, hs-CRP, fasting plasma glucose, fasting serum insulin, and serum ALT and GGT.	17
LaKind et al. (2012) [15]	The United States	Cross-sectional	Urine	ng/mL	4823	BPA continuous	0.995	0.982–1.007	Creatinine, age, gender, ethnicity, education, income, smoking, drinking, BMI, waist circumference, hypertension, total cholesteroland family history.	15
Kim & Park (2013) [16]	Korea	Cross-sectional	Urine	ng/mL	99 / 1210	BPA in quartiles Q1: < 1.36, Q2: 1.36–2.14 Q3: 2.15–3.32, Q4: > 3.32	1.71	0.89−3.26	Creatinine, age, sex, BMI, education, smoking, income and place of residence.	17
Sabanayagam et al. (2013) [17]	The United States	Cross-sectional	Urine	ng/mL	1108 / 3516	BPA in tertiles Q1: < 1.3, Q2: 1.3–3.2, Q3: > 3.2	1.34	1.03–1.73	Age (years), gender (male, female), race-ethnicity (non-Hispanic whites, non-Hispanic blacks, Mexican Americans, others), education categories (below high school, high school, above high school), smoking (never, former, current), alcohol intake (never, former, current), body mass index (normal, overweight, obese), physical inactivity (absent, present), mean arterial blood pressure (mm of Hg), C-reactive protein and total cholesterol/HDL ratio	13
Casey & Neidell et al. (2013) [18]	The United States	Cross-sectional	Urine	ng/mL	487 / 4658	BPA continuous	1.065	0.973–1.166	Age, sex, urinary creatinine concentration, race/ethnicity, income, smoking, BMI, waist circumference, veteran/military status, citizenship status, marital status, household size, pregnancy status, language at subject interview, health insurance coverage, employment status in the prior week, consumption of bottled water in the past 24 h, consumption of alcohol, annual consumption of tuna fish, presence of emotional support in one’s life, being on a diet, using a water treatment device, access to a routine source of health care, vaccinated for Hepatitis A or B, consumption of dietary supplements (vitamins or minerals), and inability to purchase balanced meals on a consistent basis.	16
Sun et al. (2014) [19]	The United States (NHS)	Case-control	Urine	μg/L	394 / 787	BPA in quartiles Q1: < 1.0, Q2: 1.0–1.5, Q3: 1.5–2.7, Q4: > 2.7	0.98	0.6–1.61	Age, ethnicity, fasting status, time of sample collection, menopausal status, use of hormone replacement therapy (NHSII), urinary creatinine levels, smoking, postmenopausal hormone use (NHS), oral contraceptive use (NHSII), physical activity, drinking, family history of diabetes, history of hypercholesterolemia or hypertension, Alternative Health Eating Index score, BMI	15
	The United States (NHS II)	Case-control	Urine	μg/L	577 / 1154	BPA in quartiles Q1: < 1.0, Q2: 1.0–1.5, Q3: 1.5–2.7, Q4: > 2.7	2.08	1.17−3.69		
Ahmadkhaniha et al. (2014) [20]	Iran	Case-control	Urine	μg/L	119 / 239	BPA in two groups based on the median (< 0.85 and ≥ 0.85 μg/L)	57.6	21.1−157.05	Age, sex, BMI, hypertension, serum triglyceride level, serum cholesterol level, serum creatinine (smoking and consumption of sugared drinks in plastic bottles or canned food in two past weeks were exclusion criteria)	15
Andra S.S. et al. (2015) [21]	The United States	Cross-sectional	Urine	ng/mL	20/ 131	BPA continuous	0.77	0.24–2.04	Age, sex, BMI, fasting status, smoking, alcohol use, physical activity and family history	18
Aekplakorn W et al. (2015) [22]	The Thailand	Cross-sectional	Serum	ng/mL	23 / 2558	BPA in quartiles Q1: < 1.0, Q2: 1.0–2.0, Q3: 2.0–3.7, Q4: > 3.7	1.88	1.18–2.99	Age, sex, urinary creatinine, race, education, smoking, physical activity, dietary energy intake and survey wave	17
Bi Y. et al. (2016) [23]	China	prospective	Urine	ng/mL	241 / 2209	BPA in quartiles	0.78	0.53–1.16	Age, sex, family history of diabetes, BMI (for weighted GRS), and further for smoking status, systolic blood pressure, diastolic blood pressure, lg (total cholesterol), lg (triglycerides), fasting plasma glucose,and lg (urinary creatinine) for BPA.	16
Shu et al. (2018) [24]	China	Case-control	Serum	ng/mL	232 / 464	BPA in tertiles	0.93	0.41–2.13	Age, sex, BMI, exercise, current smoking, systolic blood pressure, diastolic blood pressure, fasting plasma glucose, 2-h plasma glucose in oral glucose tolerance test, total cholesterol, triglyceride, high-density lipoprotein cholesterol and low-density lipoprotein cholesterol .	15

**Fig. 2:**
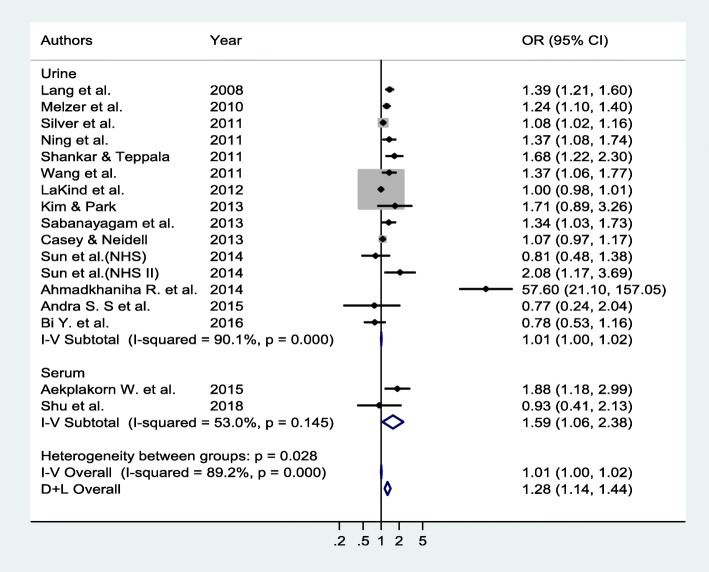
Forest plot according to sample type

**Fig. 3:**
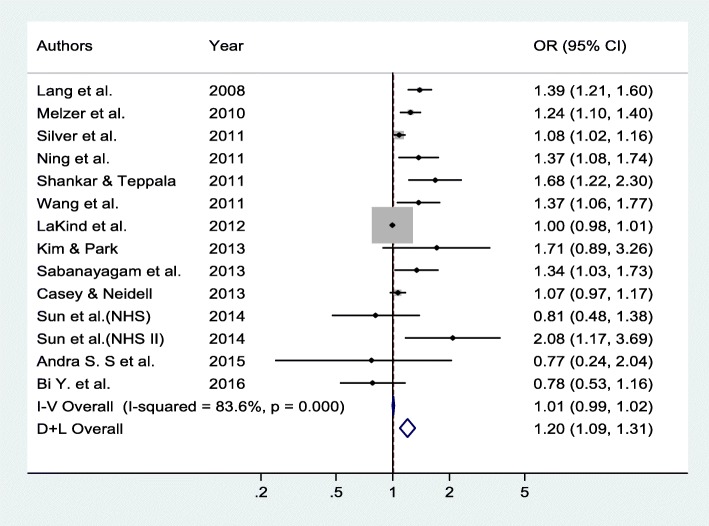
Forest plot after exclusion of studies with serum BPA levels and high heterogeneity

BPA exposure was positively associated with the risk of T2DM (Fig. 2). The pooled OR of the random-effects model was 1.28 (95% CI, 1.14–1.44). Figure 3 presents the forest plot of sensitivity analysis after three studies were excluded, one for exhibiting highly heterogeneous results (OR 57.60; 95% CI 21.10–157.05) [20] and two for using serum BPA concentrations [22, 24].

**Fig. 4:**
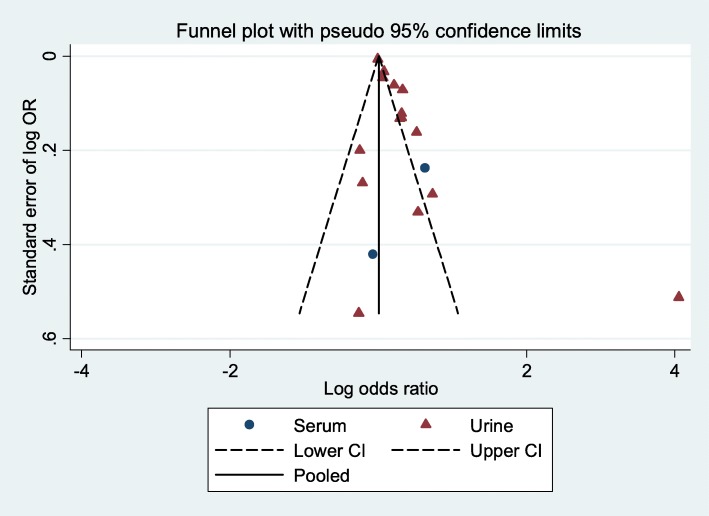
Funnel plot according to sample type

In Fig. 4 and Additional file 2: Figure S2, the funnel plot shows publication bias in the meta-analysis. Of the studies used in 16 final meta-analysis, only five were found to have no bias, four using urine BPA and one with serum BPA.

## Discussion

In this meta-analysis, we observed that the exposure of BPA was associated with an increased risk of T2DM. Both urine and serum BPA levels were positively associated with the risk of T2DM. The results of this study showed pooled OR of 1.28 (95% CI 1.14–1.44).

Previous studies have identified that the association between urinary BPA levels and T2DM may be biologically feasible. For example, BPA, an estrogen agonist that acts as an endocrine hormone disruptor, has been shown to be involved in several mechanisms of diabetes development including glucose homeostasis, obesity, insulin resistance, beta-cell dysfunction, inflammation, and oxidative stress [30]. BPA binding to estrogen receptors (ER) at concentrations at the physiological range or below can disrupt the pancreatic islets of Langherans, which are an essential tissue responsible for glucose metabolism [31]. BPA binding to pancreatic islet cells can induce impaired insulin or glucagon secretion, leading to an insulin-resistant state. In animal studies, adult mice exposed to low-dose BPA displayed both hyperinsulinemia and insulin resistance that are associated with pancreatic beta-cell dysfunction [32]. BPA can also act on peripheral insulin-sensitive tissues like muscle, liver, and adipose tissue [31]. Several in-vivo studies reported that BPA exposed mice showed decreased levels of circulating adiponectin as well as dysregulation of insulin signaling in skeletal muscle and liver. The mice also showed increased levels of pro-inflammatory cytokines, such as interleukin-6 and tumor necrosis factors, which favor the development of insulin resistance [33]. Additionally, BPA has an obesogen effect resulting in the development of obesity and metabolic disorders. Sheep exposed to BPA during the prenatal period became overweight, experienced an increase in adipocyte mass, and in insulin resistance [34–36]. The induction by BPA of the insulin resistance that precedes T2DM is mainly seen when humans and animals are in a rapid growth phase. Some studies have shown that BPA exposure during pregnancy or childhood causes metabolic disorders in both humans and animals [37, 38]. However, further studies are needed to clarify the complete mechanisms of BPA exposure and T2DM risk.

Previous meta-analyses, have not implemented the dose-response analytic method used in this study to determine the relationship between BPA exposure and the risk of T2DM. A significant dose-response relationship was found between urinary BPA concentrations (mg/dL) and T2DM risk. In addition, subgroup analysis was performed according to the type of sample (urine or serum), and the type of study (cross-sectional, case-control and prospective study). Moreover, quality assessment methods were implemented to remove any irrelevant studies and to improve the validity of the meta-analysis. The current study considered only diabetes as a primary outcome variable as was the case with the final sixteen articles used for the meta-analysis. Despite the fact that diabetes mellitus is an important risk factor for cardiovascular disease, our study will focus specifically on T2DM, and as a result will be more focused and statistically significant than previous studies.

There were several limitations to the conduct and analysis in this study that must be considered. First, because of the limited number of cohort studies investigating the relationship between BPA exposure and T2DM risk that have been conducted, this meta-analysis included only thirteen cross-sectional, two case-control studies and one prospective studies. The inclusion of additional studies are required to validate and confirm these results. Second, this meta-analysis included fourteen studies which used spot urinary BPA concentrations and two study that used serum BPA concentrations as a surrogate marker of BPA exposure. It is unclear whether spot urinary BPA concentrations could accurately reflect the long term exposure level of BPA in individuals. Spot urinary BPA concentrations are the most commonly used method to assess BPA exposure levels because of it is short half-life and the convenience of the measurement method [11, 20]. Although some studies have demonstrated that spot urine samples can reasonably predict long-term exposures in adults [39, 40], the validity of such results still needs to be proven. Some recent epidemiological studies used serum BPA concentrations to investigate the health effects of BPA [22, 40]. In these studies, the authors explained that serum BPA could be an appropriate surrogate for BPA exposure because serum BPA reflected the true levels of active BPA [22]. There is not sufficient information to determine the most suitable method for measuring BPA concentrations (e.g. spot urinary BPA concentrations, 24-h urinary BPA concentrations, serum BPA concentration) that accurately reflect the level of BPA exposure. Third, although linear relationships between BPA exposure and risk of T2DM were tested in this meta-analysis, several studies have suggested inverted U-shape or non-linear relationships [19, 21, 22]. To clarify this complex dose–response relationship, more detailed research is required. Fourth, a random effects model was implemented after performing statistical heterogeneity tests because of the significant effect that heterogeneity in inclusive studies could have on the meta-analystic results.

## Conclusions

In conclusion, this meta-analysis demonstrated that BPA concentrations measured in urine or serum is positively associated with T2DM risk. Furthermore, prospective cohort studies, including carefully collected data about the dietary sources of BPA exposure and potential confounding, will help clarify the role of BPA in the pathogenesis of diabetes.

## Additional files

Additional file 1: PRISMA 2009 Checklist. Preferred report items for systematic review and meta-analysis were identified through a checklist. (DOC 64 kb)

Additional file 2: **Figure S1.** Funnel plot with egger. **Figure S2**. GLST. These are additional sub-analysis results. (PPTX 44 kb)

## Abbreviations

BPA: Bisphenol A
CI: Confidence interval
EDC: Endocrine disrupting chemicals
OR: Odds ratio
T2DM: Type 2 diabetes mellitus
