# Supplementary figures and images for: Bisphenol A exposure and type 2 diabetes mellitus risk: a meta-analysis
Source: BMC Endocr Disord. 2018 Nov 6;18:81. doi: 10.1186/s12902-018-0310-y (PMC6219165; doi:10.1186/s12902-018-0310-y)

## Slide 1
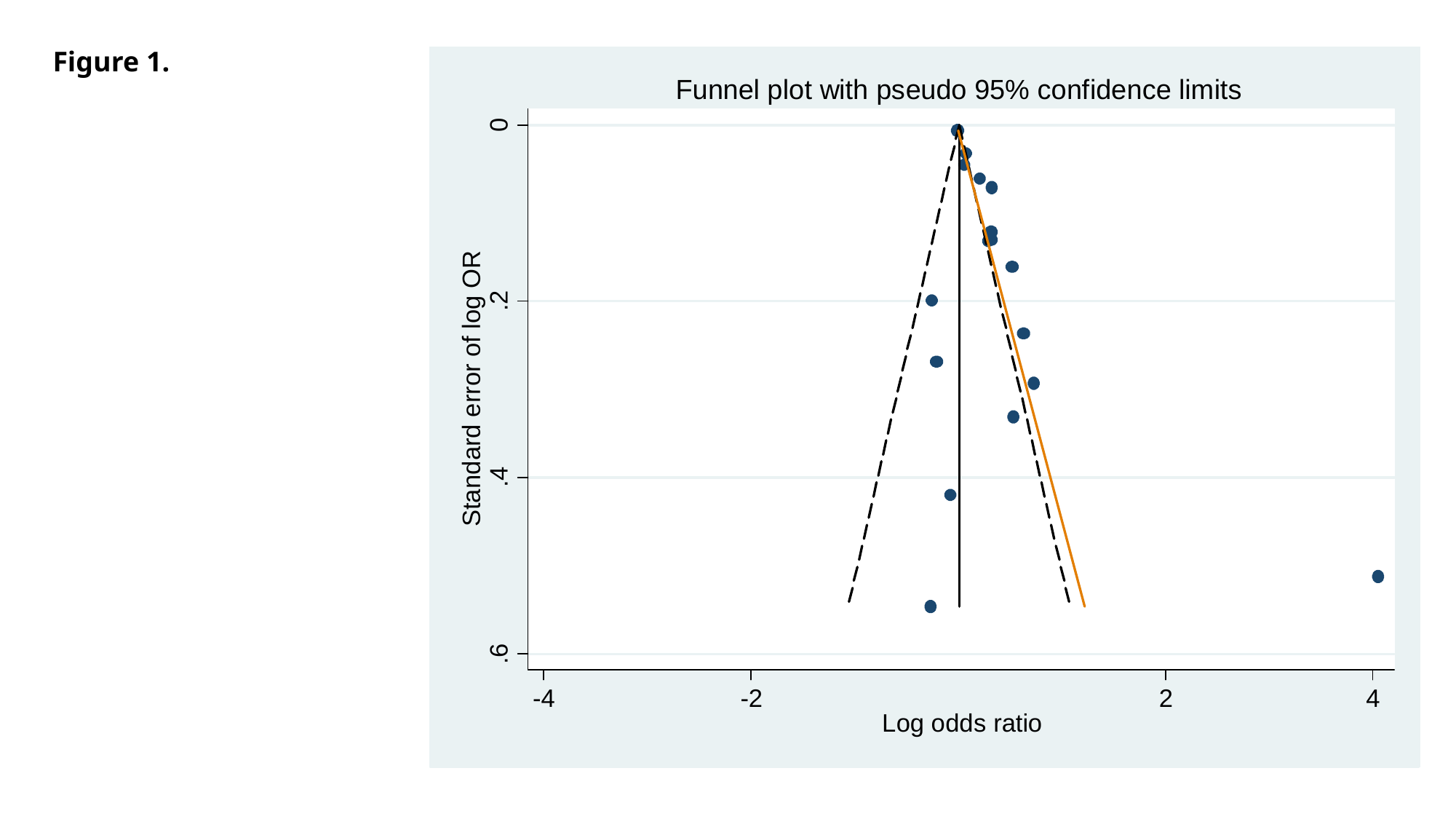

Figure 1.

## Slide 2
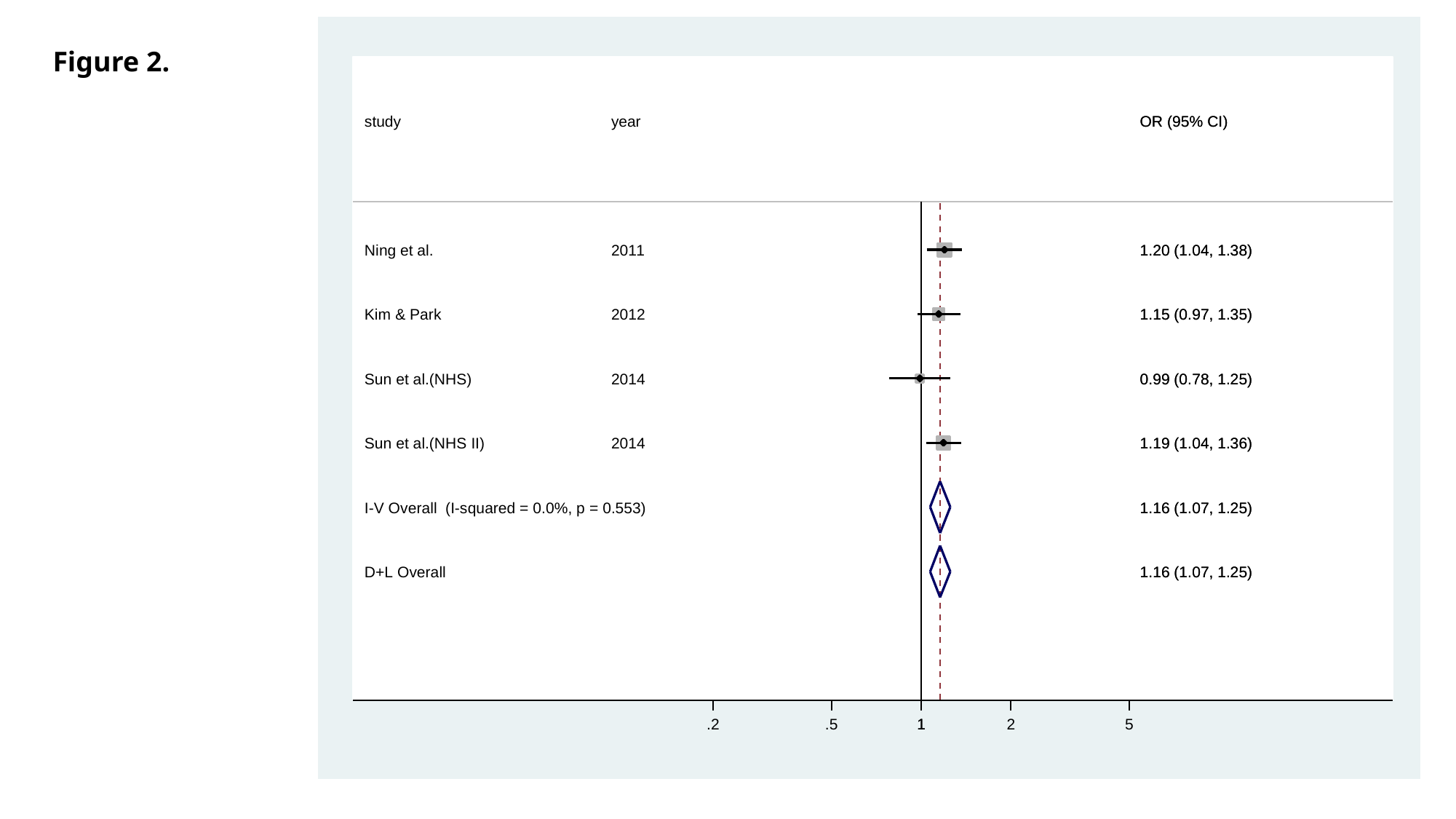

Figure 2.

Supplement: Supplementary file 2 — Figure S1. Funnel plot with egger. Figure S2. GLST. These are additional sub-analysis results. (PPTX 44 kb) [file 12902_2018_310_MOESM2_ESM.pptx]
